# High levels of cellular proliferation predict pseudoprogression in glioblastoma patients

**DOI:** 10.3892/ijo.2011.1260

**Published:** 2011-11-11

**Authors:** HENRI-BENJAMIN POULEAU, NILOUFAR SADEGHI, DANIELLE BALÉRIAUX, CHRISTIAN MÉLOT, OLIVIER DE WITTE, FLORENCE LEFRANC

**Affiliations:** 1 Department of Neurosurgery, Université Libre de Bruxelles (U.L.B.), Brussels, Belgium; 2 Department of Radiology, Université Libre de Bruxelles (U.L.B.), Brussels, Belgium; 3 Emergency, Hôpital Erasme, Université Libre de Bruxelles (U.L.B.), Brussels, Belgium; 4 Laboratoire de Toxicologie, Faculté de Pharmacie, Université Libre de Bruxelles (U.L.B.), Brussels, Belgium

**Keywords:** cellular proliferation, glioblastoma, Ki67, pseudo-progression, radiochemotherapy

## Abstract

Radiochemotherapy (RT) with concomitant followed by monthly temozolomide (TMZ) chemotherapy is the gold standard for the treatment of glioblastoma (GBM) patients. GBM patients can experience transient radiological deterioration after concurrent RT/TMZ that stabilizes or even resolves after additional cycles of adjuvant TMZ, a phenomenon defined as radiological pseudoprogression. The aim of this retrospective study was to identify a reliable marker associated with pseudoprogression processes. Patients with histologically proven newly diagnosed GBM were identified from a retrospective database between 2005 and 2009. Predictive factors for pseudoprogression were analyzed from clinical, radiological and biological data. Of the 130 analyzed patients, 63 underwent RT/TMZ treatment followed by cycles of TMZ and were evaluated for radiological responses every two months by magnetic resonance imaging. Early progression was confirmed in 52% (33/63) of the patients, and, within this group, 21% (7/33) displayed evidence of pseudo-progression. The predictive factors were evidenced in terms of clinical or radiological findings. In GBM patients, the level of cellular proliferation (Ki67 indices) emerged as a statistically significant prognostic marker for distinguishing pseudoprogression from actual progression. Our observation, suggesting that GBM associated with a high level of cellular proliferation may differentiate tumor progression from pseudoprogression, warrants further investigation in a large multi-center prospective study.

## Introduction

Gliomas are the most common primary brain tumors in adults, representing more than 50% of all brain tumors. Among them, glioblastoma (GBM) is the most biologically aggressive type, accounting for approximately 50% of all glial tumors, and is associated with the worst prognosis (1). Malignant gliomas are associated with dismal prognoses because glioma cells can actively migrate within the brain, often traveling relatively long distances, and making them elusive targets for effective surgical management (1,2). After the surgical resection and the adjuvant treatment of a glioma, the residual tumor cells peripheral to the excised dense cellular tumor core give rise to a recurrent tumor. In more than 90% of cases, this tumor develops immediately adjacent to the resection margin or within 2 cm of the resection cavity (2). Furthermore, studies have demonstrated that invasive glioma cells show a decrease in their rate of proliferation and a relative resistance to apoptosis when compared to the highly cellular tumor core, which may play roles in their resistance to conventional pro-apoptotic chemotherapy and radiotherapy (2). The multimodal standard treatment protocol for GBM consists of surgery followed by concurrent radiotherapy and chemotherapy and finally adjuvant chemotherapy with the alkylating drug TMZ (1,3,4). It has been demonstrated that a more extensive surgical resection is associated with a longer life expectancy for both low- and high-grade gliomas (5,6). The 5-year survival rate is 9.8% with the combination of radio- and TMZ therapy compared to 1.9% with radiotherapy alone (3).

This treatment regimen is often associated with significant lymphopenia, thrombocytopenia, and progressive blood-brain-barrier dysfunction that can result in clinical and radiologic deterioration without true tumor progression (7). Recently, there has been increased awareness of progressive and enhancing lesions and peritumoral edema visible by magnetic resonance imaging (MRI) immediately after RT/TMZ treatment (8–10). Although in some cases, these changes reflect tumor growth due to the treatment resistant nature of GBM, they can remain stable or diminish over time and may be a treatment effect, referred to as pseudoprogression. Enlargement of the lesion, even during the first follow-up MRI, is frequent, occurring in close to 50% of the patients (10,11). Therefore, TMZ treatment is not abandoned on the basis of seemingly discouraging imaging results within the first months following RT/TMZ. However, the percentage of true progression within the early progression cohort is highly variable in the published literature, ranging from 35% to >80% (9,11–16).

Therefore, there is a need for novel imaging techniques or biochemical markers that can better distinguish pseudoprogression from true progression to avoid unnecessary and potentially harmful surgical interventions or time lost, as TMZ treatment becomes ineffective in almost half of radiologically progressive GBM patients.

The aim of this retrospective study was to potentially correlate clinical, radiological and pathological data from GBM patients with the existence of pseudoprogression.

## Patients and methods

### Patients

All patients receiving RT/TMZ for newly diagnosed GBM between July 2005 and December 2009 were identified from the retrospective database of the Department of Neurosurgery at Erasme Hospital. TMZ was administered at a daily dose of 75 mg per m^2^ concurrent with radiotherapy and followed by 150–200 mg per m^2^ for five days every 28 days. Local research ethics board approval was obtained for this retrospective chart review (ref Erasme P2010/073). The clinical data collected included age, sex, extent of surgery, number of adjuvant TMZ cycles, and date of death.

**Figure 1:**
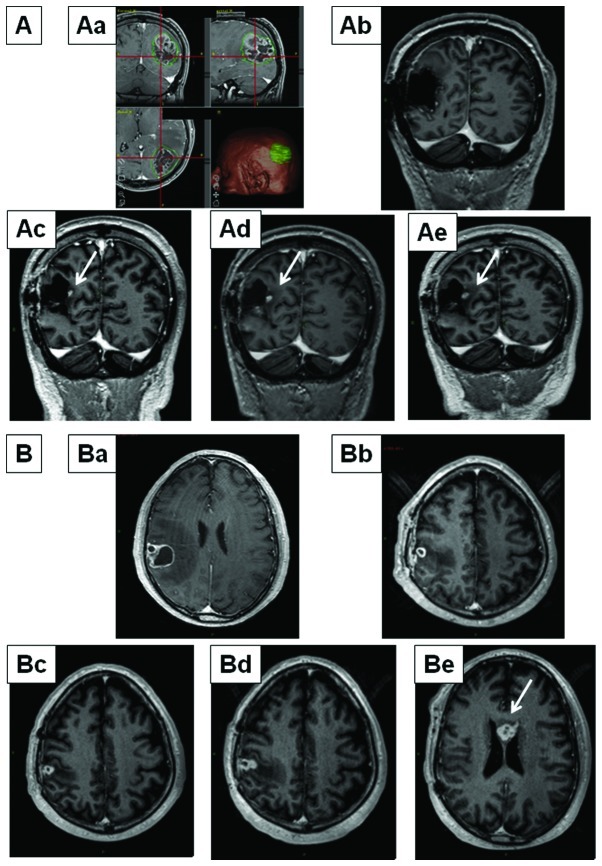
Patients with early radiological progression (progression within eight weeks after completing RT/TMZ), were further subdivided into pseudoprogression (A) and true progression (B) groups. (A) A 53-year-old man with GBM. (Aa) MR preoperative images. (Ab) Coronal T1-weighted MR image with contrast obtained 24 h after surgery revealed a macroscopically complete resection of the tumor. (Ac) Coronal T1-weighted with contrast at the same level as (Ab) obtained 1 month after the end of RT/TMZ demonstrated an enhancing lesion (white arrow). (Ad and Ae) Stabilization of the enhancing portion (white arrows) was seen on the follow-up MR images respectively after 3 and 6 cycles of TMZ adjuvant treatment. (B) A 64-year-old man with GBM. (Ba) MR preoperative images. (Bb and Bc) Axial T1-weighted MR images with contrast obtained at baseline (Bb) and 3 months after surgery (Bc). The contrast-enhancing lesion increased in size during TMZ adjuvant treatment (Bd) and a new lesion (white arrow) appeared confirming a true progression process (Be).

Patients were first radiologically categorized as early progression, which was defined as progression within 8 weeks of completing RT/TMZ. Patients with early radiological progression were further subdivided into pseudoprogression and true progression groups (Fig. 1). We defined radiological pseudoprogression as progressive enhancing lesions with peritumoral edema at MRI within eight weeks of completing RT/TMZ treatment, without clinical signs of deterioration, that stabilizes or even resolves after additional cycles of adjuvant TMZ.

Anatomopathological data and immunohistochemical markers relating to the expression of p53 protein and to the level of cellular proliferation (measured by Ki-67 index by means of the MIB-1 antibody) were analyzed.

### Statistical analyses

The descriptive statistics consisted in the use of box-and-whisker plot. In the basic box-and-whisker plot, the central box represents the values from the lower to upper quartile (25–75 percentile, interquartile range, IQR). The middle line represents the median. The whiskers represent the lowest datum still within 1.5 IQR of the lower quartile, and the highest datum still within 1.5 IQR of the upper quartile. The categorical data were compared using a χ^2^ test and continuous data using a Mann-Whitney non-parametric test. A p<0.05 was considered significant. The analyses were performed using Statistix 9^®^ (Analytical Software, Tallahassee FL, USA).

## Results

### A total of 130 patients with newly histopathologically confirmed GBM were identified

**Figure 2:**
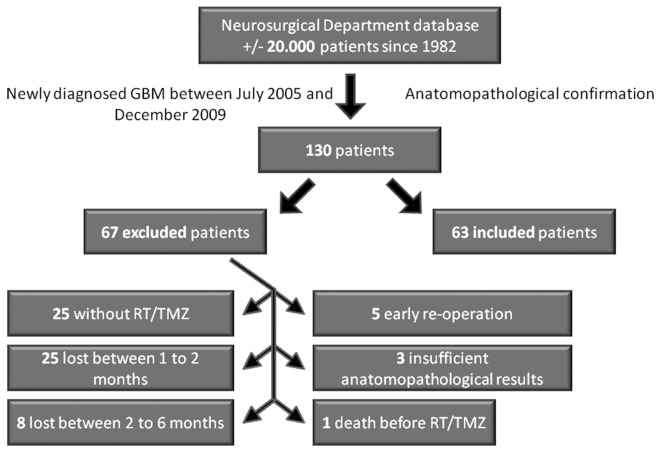
Patient selection.

**Figure 3:**
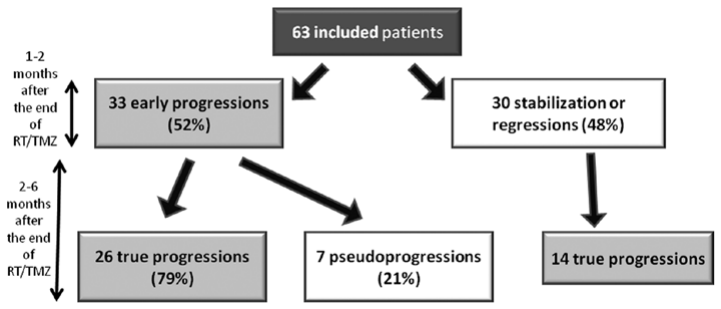
Of the early progressions 21% is pseudoprogression.

Sixty-seven patients were excluded: 25 did not receive RT/TMZ, 25 were lost to follow-up at 1–2 months and 8 at 2–6 months following combined RT/TMZ, 5 were re-operated early on, 3 showed insufficient anatomopathological markers and 1 died before RT/TMZ (Fig. 2). Sixty-three patients between the ages of 27 and 78 were therefore selected for the study. Demographic data are shown in Table I. Thirty-three of 63 (52%) patients showed early progression (progression within eight weeks after completing RT/TMZ), of which 7 (21%) were identified as showing pseudoprogression, and 26 (79%) were identified as true progression (Fig. 3). In the group with no early progression (30 patients), 14 (47%) followed a pejorative evolution between two to six months following the end of the concomitant RT/TMZ treatment (Fig. 3).

**Figure 4:**
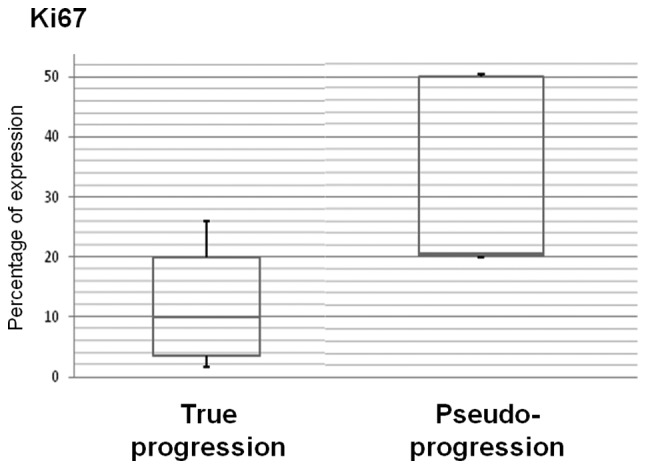
Levels of cellular proliferation by means of Ki67 antigen labeling. All GBM showing a pseudoprogression phenomenon also showed a level of cellular proliferation ≥20%. Data are presented in box-and-whisker plots.

The evaluation of p53 overexpression in GBM tissue was categorized into four classes: no, low, intermediate and high overexpression. While levels of p53 expression were of no predictive value in the identification of pseudoprogression (data not shown), the Ki67 index, related to the level of cellular proliferation, was predictive (Fig. 4). Indeed, the median level of cellular proliferation within the group of pseudoprogression GBP patients was significantly (p=0.0016) higher [20% (IQR 20–50%)] compared to the group of GBM patients associated with true progression [10% (IQR 3.5–20%] (Fig. 4).

## Discussion

The diagnosis of pseudoprogression is highly relevant to the practice of neuro-oncology. In a phase III prospective study, De Wit and collaborators (17) showed that radiotherapy alone could bring about pseudoprogression in three out of 32 GBM patients (9%). This finding could be one possible explanation for the worsening of imaging studies and clinical deterioration observed following the completion of RT/TMZ. The incidence of pseudoprogression in the early progression patient population reported in the literature varies from 12 to 64% of pseudoprogression cases (8–16). In our study, seven of the 63 patients studied developed pseudoprogression, representing 11% of the patients receiving RT/TMZ treatment and 21% (7/33) of the patients with signs of early progression.

Suspicion of pseudoprogression may influence a clinician’s recommendation to continue with standard adjuvant TMZ chemotherapy rather than beginning a second line therapy for recurrence. Imaging changes consistent with pseudoprogression commonly persist for up to three months following the completion of RT/TMZ and occasionally are persistent for longer periods.

In the pivotal EORTC-NCIC-CTG trial, up to 20% of patients did not receive maintenance TMZ therapy, usually due to deterioration in post-treatment imaging (4). However, TMZ, given as maintenance therapy for at least six months in appropriate patients, is one of the most active agents currently approved for GBM. The therapeutic benefits of TMZ are due to the fact that it induces double strand DNA breaks via the generation of methyl-guanosine (18) concomitantly with sustained autophagy-related processes (19,20), with both of these effects resulting in the apoptosis of GBM cells (21). TMZ also displays anti-angiogenic effects (22). In contrast, TMZ treatment of GBMs can lead to the emergence of TMZ-resistant tumors, at least at the experimental level (23,24).

Therefore, the occurrence of pseudoprogression following standard therapy for GBM raises important issues related to the determination of disease progression, the optimal timing and methods to judge treatment efficacy, when to recommend second line or experimental therapy, and how to evaluate new agents administered ‘on the back of’ RT/TMZ.

The biology of pseudoprogression is not clear, and several hypotheses are found in the literature (8,9,11,17,25). While Chamberlain *et al* (26) have demonstrated that some patients develop early radionecrosis following RT/TMZ, the issue of pseudoprogression is different. Pseudoprogression likely involves early changes to the vascular endothelium and the blood-brain-barrier associated with vasogenic edema; however, the precise mechanism remains complex and poorly understood (8). Combined with the radiotherapy effect, TMZ, which induces cellular replication arrest in the G2/M cell cycle phase (the phase most sensitive to radiotherapy) and increases the number of DNA breaks in GBM cells (18), could have a role in the pseudoprogression phenomenon. The increase in contrast enhancement during pseudoprogression could also be due to cellular hypoxia secondary to the combined treatment (25). Cellular hypoxia leads to dysregulation of the expression of several molecules including hypoxia-inducible factor-1α (HIF-1α) (25). In the absence of HIF-1α regulation, DNA promoter regions are activated, leading to an increase in the transcription of hypoxia response elements (HREs) (25). These HREs conduct the transcription of more than one hundred genes, leading to an increase in the synthesis of vascular endothelial growth factor (25). With the goal being to help hypoxic cells, these processes increase vascular permeability and therefore lead to an increase in contrast enhancement and angiogenesis (25).

Future studies will likely take advantage of developments in modern MRI-based vascular permeability, flow imaging, spectroscopy and PET scanning (27,28) and in alternative end-points and response criteria developed by an international working group (29) to elucidate the nature and timing of these changes. A recent study suggests that relative cerebral blood volume measured by dynamic susceptibility-weighted contrast enhanced perfusion MRI has an impact on the predictability of pseudoprogression in patients with GBM (30). Perhaps an imaging tool can be developed to assist the clinician to determine the difference between a patient with a robust treatment response (conferring a survival advantage) versus a patient with disease resistance. Until then, we must be cautious with the interpretation of imaging following the treatment of GBM.

Our study suggests a statistically significant difference in the levels of cellular proliferation, observed by means of the percentage of Ki67 antigen expression, between pseudoprogression and true progression. All patients with pseudoprogression showed a GBM tumor associated with a level of Ki67 expression ≥20%. To our knowledge, this is the first time that a link between the level of cellular proliferation in GBMs and the development of a pseudoprogression phenomenon during or just after RT/TMZ has been reported. This phenomenon could be explained, at least in part, by the fact that RT/TMZ induces cell death during the replication phase of the cell cycle. Thus, this phase would show the highest level of cellular replication and the highest observed initial effects of the treatment (8,9,11,25). The study by Brandes *et al* (11) argues this point because that group has demonstrated that the level of pseudoprogression is significantly higher in the presence of MGMT (O^6^-methylguanine methyltranferase) promoter methylation compared to its absence (66 vs. 34%). The inactivation of the repairing enzyme MGMT increases the efficiency of TMZ (3,18,31). A recent study revealed that methylation-specific multiplex ligation probe amplification, an assay that permits the semi-quantitative evaluation of promoter methylation, is a useful method for predicting radiological progression versus pseudoprogression in GBM patients, and the interpretation of the results, in combination with methylation-specific polymerase chain reaction results, will provide good practical guidelines for clinical decision making regarding GBM treatment (32).

Our observation, suggesting that GBM associated with high levels of cellular proliferation may differentiate tumor progression from pseudoprogression, warrants further investigation in a prospective study with a larger number of patients. We plan also to analyze the individual versus combined prognostic information contributed by MGMT status and Ki67-related cell proliferation levels.

## Figures and Tables

**Table I:** Demographics of population.

Age	
Median (years)	59.5
<50	15 (23.8%)
50–59	16 (25.4%)
60–69	19 (30.2%)
≥70	13 (20.6%)
Gender	
Female/Male	24/39 (38.1%/61.9%)
Surgery	
Gross total	58 (92%)
Partial resection	2 (3.2%)
Open biopsy	1 (1.6%)
Stereotactic biopsy	2 (3.2%)
